# Supplementary material for: Risk assessment of debris flow disaster based on the cloud model—Probability fusion method
Source: PLoS One. 2023 Feb 2;18(2):e0281039. doi: 10.1371/journal.pone.0281039 (PMC9894451; doi:10.1371/journal.pone.0281039)
Supplement: Attachment — Submitted filename: Response to Reviewers.docx [file pone.0281039.s003.docx]

**Risk Assessment of Debris Flow Disaster Based on the Cloud Model - Probability Fusion Method**

Li Li^*^, Bo Ni, Yue Qiang, Shixin Zhang, Dongsheng Zhao, Ling Zhou

**Itemized responses to comments of academic editors and reviewers**

The authors thank the editor and the reviewers’ support for publishing our manuscript. The authors are also grateful to the editor and the reviewers for the constructive suggestions that improved the manuscript. Based on the reviewers’ comments, the manuscript has been revised, and the revisions are marked in red. Our responses to the comments are presented below, numbered for ease of reference.

**Journal Requirements:**

**1.	Comment:** Please ensure that your manuscript meets PLOS ONE's style requirements, including those for file naming. The PLOS ONE style templates can be found at

https://journals.plos.org/plosone/s/file?id=wjVg/PLOSOne_formatting_sample_main_body.pdf and

https://journals.plos.org/plosone/s/file?id=ba62/PLOSOne_formatting_sample_title_authors_affiliations.pdf

**Response:** The author thanked the editor for the reminder, which has been revised in line with journal requirements.

**2.	Comment:** Please note that PLOS ONE has specific guidelines on code sharing for submissions in which author-generated code underpins the findings in the manuscript. In these cases, all author-generated code must be made available without restrictions upon publication of the work.

Please review our guidelines at https://journals.plos.org/plosone/s/materials-and-software-sharing#loc-sharing-code and ensure that your code is shared in a way that follows best practice and facilitates reproducibility and reuse.

**Response:** The author thanks the editor for the reminder that the code has been uploaded as a supplementary file.

**3.	Comment:** We note that the grant information you provided in the ‘Funding Information’ and ‘Financial Disclosure’ sections do not match.

When you resubmit, please ensure that you provide the correct grant numbers for the awards you received for your study in the ‘Funding Information’ section.

**Response:** The author thanked the editor for the reminder, which has been revised in line with journal requirements.

**4.	Comment:** Thank you for stating the following financial disclosure:

~

Please state what role the funders took in the study. If the funders had no role, please state: ""The funders had no role in study design, data collection and analysis, decision to publish, or preparation of the manuscript."" If this statement is not correct you must amend it as needed.

Please include this amended Role of Funder statement in your cover letter; we will change the online submission form on your behalf.

**Response:** The author thanks the editor for the reminder, which has been modified in cover letter.

**5.	Comment:** Thank you for stating the following in the Acknowledgments Section of your manuscript:

~

We note that you have provided funding information that is not currently declared in your Funding Statement. However, funding information should not appear in the Acknowledgments section or other areas of your manuscript. We will only publish funding information present in the Funding Statement section of the online submission form.

Please remove any funding-related text from the manuscript and let us know how you would like to update your Funding Statement. Currently, your Funding Statement reads as follows:

~

Please include your amended statements within your cover letter; we will change the online submission form on your behalf.

**Response:** The author thanks the editor for the reminder, which has been modified in cover letter.

**6.	Comment:** In your Data Availability statement, you have not specified where the minimal data set underlying the results described in your manuscript can be found. PLOS defines a study's minimal data set as the underlying data used to reach the conclusions drawn in the manuscript and any additional data required to replicate the reported study findings in their entirety. All PLOS journals require that the minimal data set be made fully available. For more information about our data policy, please see http://journals.plos.org/plosone/s/data-availability.

Upon re-submitting your revised manuscript, please upload your study’s minimal underlying data set as either Supporting Information files or to a stable, public repository and include the relevant URLs, DOIs, or accession numbers within your revised cover letter. For a list of acceptable repositories, please see http://journals.plos.org/plosone/s/data-availability#loc-recommended-repositories. Any potentially identifying patient information must be fully anonymized.

Important: If there are ethical or legal restrictions to sharing your data publicly, please explain these restrictions in detail. Please see our guidelines for more information on what we consider unacceptable restrictions to publicly sharing data: http://journals.plos.org/plosone/s/data-availability#loc-unacceptable-data-access-restrictions. Note that it is not acceptable for the authors to be the sole named individuals responsible for ensuring data access.

We will update your Data Availability statement to reflect the information you provide in your cover letter.

**Response:** The author thanks the editor for the reminder, which has been modified in cover letter.

**7.	Comment:** PLOS requires an ORCID iD for the corresponding author in Editorial Manager on papers submitted after December 6th, 2016. Please ensure that you have an ORCID iD and that it is validated in Editorial Manager. To do this, go to ‘Update my Information’ (in the upper left-hand corner of the main menu), and click on the Fetch/Validate link next to the ORCID field. This will take you to the ORCID site and allow you to create a new iD or authenticate a pre-existing iD in Editorial Manager. Please see the following video for instructions on linking an ORCID iD to your Editorial Manager account: https://www.youtube.com/watch?v=_xcclfuvtxQ

**Response:** The author thanks the editor for the reminder that the ORCID iD has been bound to the corresponding author.

**8.	Comment:** We note that Figures 1 and 2 in your submission contain map images which may be copyrighted. All PLOS content is published under the Creative Commons Attribution License (CC BY 4.0), which means that the manuscript, images, and Supporting Information files will be freely available online, and any third party is permitted to access, download, copy, distribute, and use these materials in any way, even commercially, with proper attribution. For these reasons, we cannot publish previously copyrighted maps or satellite images created using proprietary data, such as Google software (Google Maps, Street View, and Earth). For more information, see our copyright guidelines: http://journals.plos.org/plosone/s/licenses-and-copyright.

**Response:** The author thanks the editor 's reminder, has deleted Fig. 1 and Fig. 2 and made a text description.

**Reviewer #1:**

The manuscript addresses evaluating the risk of debris flow disasters based on the cloud model and the Monte Carlo method. The topic is important. However, there are some problems in the manuscript. Please take into consideration of the following comments to improve your manuscript for a major revision.

**1.	Comment:** The language in the whole manuscript needs improvement. E.g., the last sentence in ‘Abstract’.

**Response:** The reviewer’s comment is indeed and the authors thank the reviewer for this comment. The author has improved the grammar of the whole manuscript.

**2.	Comment:** There are only 3 sentences in ‘Abstract’? Please improve it.

**Response:** The authors thank the reviewer for this comment. The author has improved the abstract section in lines 12 to 24.

**3.	Comment:** The work uses weight factor method. Entropy-based method is a common weight factor. I suggest the authors added some works of entropy-based weight factor for the convenience of the readership.

**Response:** The authors thank the reviewer for this comment. The author has made relevant supplements in lines 51 to 58 of the introduction.

**4.	Comment:** For example, in ‘Introduction’, please introduce some methods of entropy-based weight factor. E.g., ‘Measuring Uncertainty in the Negation Evidence for Multi-Source Information Fusion. Entropy. 2022; 24(11):1596. https://doi.org/10.3390/e24111596’.

**Response:** The reviewer’s comment is indeed and the authors thank the reviewer for this comment. The author has made relevant supplements in lines 51 to 58 of the introduction.

**5.	Comment:** Please highlight the motivation and contribution of using the cloud model and the Monte Carlo method simultaneously, e.g., in ‘Abstract’’ and ‘Introduction’.

**Response:** The reviewer’s comment is indeed and the authors thank the reviewer for this comment. The author has re-discussed the whole abstract part and made relevant supplements in lines 59 to 73 of the introduction.

**6.	Comment:** I suggest the authors introduce the flow chart of the method (Figure 8) at the beginning of the subsection, not by the end of the section. This can help the readership find the main contribution of the work easily.

**Response:** The authors thank the reviewer for this comment. The author has placed the flowchart at the beginning of Section 3.2 Model construction.

**7.	Comment:** The details of some figures should be clearly described in the main text. E.g., please explain each sub-figure of ‘Fig. 6’ in the text.

**Response:** The authors thank the reviewer for this comment. The author has made the related supplementary explanation to Fig. 1 to Fig. 6.

**8.	Comment:** The sequence number in Section 5 and 6 is not suggested. Complete paragraph is suggested.

**Response:** The authors thank the reviewer for this comment. The author has made corresponding modifications in lines 322 to 345.

**Reviewer #2:**

This paper proposes a new method for risk assessment of debris flow gully based on Monte Carlo simulation and an improved cloud model. It is interesting. I suggest minor revision.

**1.	Comment:** The English writing should be polished with help of professionals. There are some typos, grammatical errors and unsmooth expressions. For example, “literatures” should be corrected as “literature” in the 198th line on page 8.

**Response:** The reviewer’s comment is indeed and the authors thank the reviewer for this comment. The author has improved the grammar of the whole manuscript.

**2.	Comment:** On cloud model, some updated references should be commented to grasp the status of research. The following may be helpful: International Journal of Fuzzy Systems, 20(7) (2018) 2273-2300; Computer Modeling in Engineering & Sciences, 131(3) (2022) 1751-1792.

**Response:** The reviewer’s comment is indeed and the authors thank the reviewer for this comment. The author has made relevant supplements in lines 65 to 68 of the introduction.

**3.	Comment:** It would be better to add some solid comparative analyses in section 4 to explain the advantage of the proposed method of this paper.

**Response:** The reviewer’s comment is indeed and the authors thank the reviewer for this comment. The author has made relevant supplements in section 4, lines 313 to 320.

**4.	Comment:** Some future directions should be listed in section 6.

**Response:** The reviewer’s comment is indeed and the authors thank the reviewer for this comment. The author has added lines 343 to 345 in the conclusion.
